# Overview of literature on RMC and applications to Tanzania

**DOI:** 10.1186/s12978-018-0599-z

**Published:** 2018-10-03

**Authors:** Karline Wilson-Mitchell, Lucia Eustace, Jamie Robinson, Aloisia Shemdoe, Stephano Simba

**Affiliations:** 1 Midwifery Education Program, Ryerson University, 350 Victoria Street, Toronto, ON M5B 2K3 Canada; 2 Tanzanian Midwives Association, P.O. Box 65524, Muhimbili Dar Es Salaam, Tanzania; 3 Canadian Association of Midwives, 2330 Notre-Dame W., Suite 300, Montreal,, Quebec H3J 1N4 Canada

**Keywords:** Respectful maternity care, Quality improvement, Global health, Maternity care, Midwives

## Abstract

Respectful maternity care research in Tanzania continues to increase. This is an overview of the literature summarizing research based on the domains which comprise this quality of care indicator, ranging from exploratory and descriptive to quantitative measurements of birth perinatal outcomes when respectful interventions are made. The domains of respectful care are reflected in the seven Universal Rights of Childbearing Women but go further to implicate facility administrators and policy makers to provide supportive infrastructure to allay disrespect and abuse.

The research methodologies continue to be problematic and several ethical cautions restrict how much control is possible. Similarly, the barriers to collecting accurate accounts in qualitative studies of disrespect require astute interviewing and observation techniques. The participatory community-based and the critical sociology and human rights frameworks appear to provide a good basis for both researcher and participants to identify problems and determine possible solutions to the multiple factors that contribute to disrespect and abuse. The work-life conditions of midwives in the Global South are plagued with poor infrastructure and significantly low resources which deters respectful care while decreasing retention of workers. Researchers and policy-makers have addressed disrespectful care by building human resource capacity, by strengthening professional organizations and by educating midwives in low-resource countries. Furthermore, researchers encourage midwives not only to acquire attitudinal change and to adopt respectful maternity care skills, but also to emerge as leaders and change agents.

Safe methods for conducting care while addressing low resources, skilled management of conflict and creative innovations to engage the community are all interventions that are being considered for quality improvement research. Tanzania is poised to evaluate the outcomes of education workshops that address all seven domains of respectful care.

## Plain English Summary

Respectful maternity care (RMC) is a growing field of research and practice which recognizes that effective care must uphold the dignity of the birthing women. How women are treated during pregnancy and labour affects their birth experience and the health of mother and baby. Disrespectful care is a recognized problem worldwide. In low resource settings and/or areas with high mortality, such as Tanzania, disrespectful care directly impacts women’s willingness and ability to access health care and give birth with a skilled health worker present. In seeking to address maternal mortality, the focus is often on material circumstances (accessibility of care, economic circumstances); the RMC movement centers the birthing women’s experience as a key driver of birth outcomes.

The RMC movement seeks to provide common language for categorizing key themes in disrespectful care. There are seven key pillars (or domains) of RMC. Understanding how RMC impacts women’s health is essential to educate governments, health workers, and the global health industry about the importance of quality and dignity in the provision of care. Equally important, we must understand the physical, systemic, and emotional spaces that generate disrespectful care. In our personal experience of hosting RMC workshops in Tanzania, we learned firsthand from midwives and nurses about the material and temporal deprivations that shape their context. This literature review provides a broad overview of RMC issues addressed in current research and applications from our experience in Tanzania for practitioners seeking to enable dignified birth and improve birth outcomes in Sub-Saharan Africa and globally.

## Background

Respectful maternity care (RMC) is a growing field of research and practice which recognizes that to be effective, health care and assistance during pregnancy and birth must uphold the dignity of the birthing women. How women are treated during pregnancy and labour affects their birth experience and the health of mother and baby. Disrespectful care is a recognized problem worldwide. In low resource settings such as Tanzania, where there is a high maternal mortality of approximately 410–526 per 100,000 pregnancies [1, 2], disrespectful care directly impacts women’s willingness and ability to access health care and give birth with a skilled health worker. In contrast to projects which focus on material circumstances (accessibility of care, economic circumstances) to address maternal mortality; the RMC movement centers the birthing women’s experience as a key driver of birth outcomes.

The RMC movement seeks to provide common language to categorize key themes in disrespectful care. Despite varied opinions about defining ‘respectful care’, researchers and practitioners have developed a rigorous and comprehensive rubric focused on seven domains of RMC (See Table 1). These 12 domains reflect seven Universal Rights of Childbearing Women [3].

**Table 1:** Domains of respectful maternity care framework

Domain Number	Domain Description of Disrespect and Abuse	Universal Childbirth Right
1	Physical abuse (e.g., painful or embarrassing procedures without warning or unnecessarily performed)	1. freedom from harm and ill treatment
2	Non-consented care (e.g. lacks provision of information to make an intelligent decision, lack of permission or courtesy for invasive and traumatic procedures)	2. informed consent and refusal and respect for choices
3	Non-confidential care (e.g., lack of covering to provide culturally desired modesty, inappropriate sharing of client’s information, inability to track or secure patient records)	3. right to privacy and confidentiality
4	Non-dignified care (e.g. verbal abuse, psychological abuse)	4. right to dignity and respect
5	Discrimination based on specific attributes (e.g. lack of equitable maternity care regardless of group membership)	5. equality, freedom from discrimination and equitable care
6	Abandonment or denial of highest quality of care available (e.g., Provision of efficient and effective care)	6. Access to healthcare and the highest attainable level of health
7	Detention of mother or baby in facilities (e.g., for lack of payment, lack of universal access to care)	7. liberty, autonomy, self-determination, and freedom from coercion
8	Enhancing quality of physical environment and resources	6. Access to healthcare and the highest attainable level of health
9	Engaging with effective communication	4. right to dignity and respect and 5. equality, freedom from discrimination and equitable care
10	Availability of competent and motivated human resources, inability to provide continuity of care and continuity of carer (e.g., less than optimal staffing, poor fiscal management, poor recruitment and retention of personnel, loss of morale and lack of workforce job satisfaction, poor remuneration for work, poor working conditions and policies, lack of emotional and professional support for staff, lack of staff training)	6. Access to healthcare and the highest attainable level of health
11	Restriction from movement or position changes, disempowering or inequitable behaviours or policies (denying the client a culturally safe space)	4. right to dignity and respect
12	Lack of support for desires and choices (e.g. having a labour support person present at birth, declining a test or procedure, policies at the facility or governmental level that do not support the desire of mother to be accompanied by a desired family member or partner, lack of support for the special psychosocial needs of adolescents or other vulnerable populations)	2. informed consent and refusal and respect for choices and preferences even when the choice is to reject recommended community standards

Understanding how RMC impacts women’s health is essential to educate governments, health workers, and the global health industry about the importance of quality and dignity in the provision of care. Equally important, are the physical, systemic, and emotional spaces that generate disrespectful care. In our personal experience of hosting RMC workshops in Tanzania, we learned firsthand from midwives and nurses about the material and temporal deprivations that shape their context. This literature review provides a broad overview of RMC as an emerging field, current research and applications to our experience in Tanzania for practitioners seeking to enable dignified birth and improve birth outcomes in Sub-Saharan Africa and globally.

## Methods

**Fig. 1:**
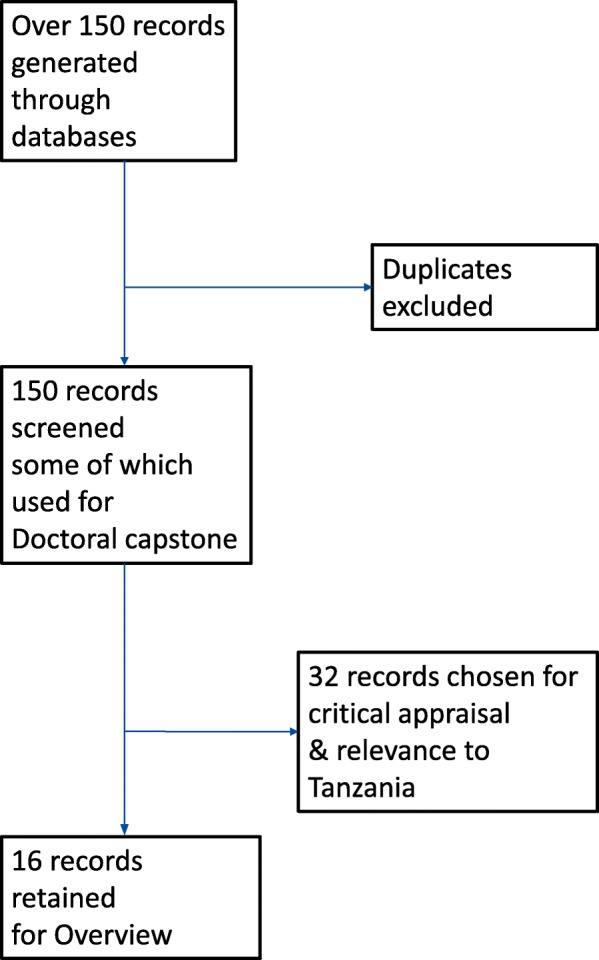
Flow chart: Literature search and selection based upon relevance

Studies and policy papers were on CINHAL, Medline, Pubmed, Proquest, Google Scholar, and Mendeley research databases as described by Arksey and O’Malley (2005) using terms: “developing countries, midwifery, life change events, global health, childbirth rights, adolescents and respectful maternity care”. A total of 32 studies and policy papers were found by using some of the elements of Arksey and O’Malley’s methods to identify research gaps in existing literature by identifying: 1. the research question, 2. Relevant studies that had an African or low-resource country-context. 3. Significant findings that we could collate, summarize and compare (see Fig. 1). Of these 32 studies, 16 are critically appraised in Table 2. Following are the three themes emerging from the overview: conceptualization and measurement, work-life experience of providers, leadership and change.

**Table 2:** Critical evidence table

No.	Author Information	Context and Sampling	Methodology (variables, analysis)	Findings and implications to practice
1.	Abuya T et al. [1]	13 facilities in Kenya (private, public and faith based) with various levels of care (hospital, nursing home, health centers and referral facilities). All with small numbers of deliveries, similar professional expertise, skills, clientele, location and fees. Nurses and midwives practices in all. Bed capacity ranged from 42 to 135 beds. Women 15–45 yr. old. *N* = 641, 85 (13.2%) ages 15-19yr.	mixed methods, 90% power what authors identify as “Quasi-experimental before-and after implementation”	1:5 women felt “humiliated” during Labour and Delivery dept. 6 categories of disrespect and abuse reported. Multiparous women 3× more likely to be detained for lack of payment, 5X more likely bribe. Study measured 10% decrease in disrespect and abuse.
2.	Bohren MA et al. [19]	Mixed methods systematic review of 65 studies, 34 countries. Developed and Developing countries included (included the studies of Kruk et al., Sando et al., and McMahon all of which included sampling in Tanzania in which self-reports were used for gathering data.	PubMed, CINAHL, and Embase databases and grey literature were searched and synthesized thematically using CERQual tool.	Key behaviours were identified in Tanzania as disrespectful (neglectfulness, preoccupation with other tasks, discrimination due to HIV status, lack of privacy, lack of consent for internal cervical exams, detention of mother or baby for lack of payment, unclear fee structures. “Mistreatment” is defined as involving the health care system and facility policies
3.	Bowser D et al. [3]	Desktop review of the published and gray literature, individual interviews with nine expert informants and a structured group discussion. Tanzania was one of 19 countries in both the Global South and North studied.	Web-based search yielding 70 peer-reviewed articles with terms: abusive care, disrespectful care, dignified birth, caring behavior, humanization of childbirth, discrimination during childbirth, stigma, detention, neglect, accountability, human rights and childbirth, health workers for change, empowerment, redress, health systems and childbirth, quality of care, barriers to treatment of obstetric emergencies, and women‘s perceptions of maternal care (150+ documents references provided by key informants (journal articles, book, reports).	A model was presented which describes the relationships between contributors or deterrents and disrespect and abuse in childbirth. These include individual, national policies and human rights, governance and leadership, service delivery and providers, financial access, geographic access and cultural birth preferences, and skill level of workers. The authors concluded that there is a connection between the woman’s autonomy and empowerment with disrespect was made.
4	Bhutta ZA et al. [4]	Rural district of Southern Pakistan, Feb 2006 to March 2008.	16- cluster randomised trial. Intervention was the use of trusted Lady Health Workers trained in culturally sensitive prenatal education.	63% (4428) of the planned group educational sessions were delivered and 2943 neonates (24%) received home visits in their catchment villages. The stillbirth rate was 39.1 per 1000 births compared to the control of 48.7. RR 0,79, 95% CI with *p* = 0.006. NMR was 43 per 1000 compared to the control of 49.1 (RR = 0.85, CI 0.76–0.96, *p* = 0.02. Although not stated, the cultural sensitivity and relationship of trust between the mother and LHW was significant. The curriculum was standard, however the exact content, delivery and behaviours were not described.
5	Duysburgh E et al. [9]	Rural Burkina Faso, Ghana and Tanzania. Tanzania’s Builsa and Kassena-Nankana districts with populations of > 30,000 and access to emergency services at the facilities sampled. In communities where 95.7% of pregnant women receive care from skilled providers.	Non-randomized intervention study. QUALMAT project Was implemented at provider level to measure the existing gap between ‘knowing what to do’ and ‘doing what you know’. Two kinds of interventions are planned: (i) performance-based incentives to increase health workers’ motivation and (ii) computer-assisted clinical decision support, to improve compliance with clinical guidelines. Exit interviews, health facility surveys and retroactive chart review.	Counselling, health education practice, laboratory investigations, equipment for vacuum assisted birth, maternal and newborn assessment and partograph use were all deficient. Rectifying these deficiencies could easily reduce MMR and NMR.
6	Hanson C et al. [14]	Rural Southern Tanzania, 226,000 households. This survey reportedly captures higher mortality rates than the facility-based MMR published by the Ministry of Health Community Development Gender Elderly and Children (MoHCDGEC)	Census data using a georeferenced household survey measuring pregnancy-related mortality ratio (number of pregnancy-related deaths reported by the household head)	60% of the mothers who lived less than 5 km from the birthing facility reportedly died secondary to poor quality care during their hospital birth with an MMR of 111 direct deaths per 100,000 livebirths.
7	Kidanto HL et al. [15]	University teaching hospital in Dar es Salaam Tanzania	Criteria-based audits (CDA) were performed to determine the contributing factors when Apgar scores for newborns were less than 7 at the 5th minute. 389 eclampsia cases audited initially and 88 cases in the re-audited for evidence-based management of eclampsia.	Providing access to the highest quality care available is one of the measurement criteria for respectful maternity care and CBA is considered a feasible method for identifying problems with quality of care. Poor performance improved when re-audited but it was noted that poor documentation and ineffective staff utilization persisted. Inexpensive recommendation could help the facility reach targeted goals.
8	Kruk ME et al. [13]	1203 were sampled out of 1322 eligible women (91% response rate). Rural western Tanzania. Most were married, ethnically 3 ethnically similar groups. Facility births account for only 1/3 of all births.	Population-based discrete choice experiment to determine factors that influence choice of birthplace. Choice A was 1 h away, care cost 500 shillings, with a doctor who neither smiled nor listened carefully and was not always available. Choice B was 3 h away, cost 3000 shillings, the nurse smiled and listened carefully and was always available. Both A and B provided free transport. Choice C meant choosing neither A nor B facility.	There was a high coefficient ratio for predicting which facility would be preferred by the women, however the provider attributes provided were minimal. Other relevant factors such as availability of drugs and equipment, provider type considered after data collection. The author concludes that facility and provider attributes were contributing to the high rates of out of facility births and possibly the use of traditional birth attendants and avoidance of the disrespectful care received at the facilities.
9	Kujawski S et al.	Tanga Region, Tanzania, 8 facilities in Korogwe and Muheza Districts, both government and non-governmental owned hospitals.	Cross sectional study, structured survey measuring satisfaction. Univariate statistics.	1388 women participated in the survey (67% response rate). Detailed description of the sample (39% primiparous, 20.87% with secondary education or higher, households, 25.42% with access to electricity and 85.21% had access to a mobile phone. There was a correlation between respectful care, perceived quality of care, intention to deliver at that facility again in future were all related to perceptions of satisfaction. RMC was not clearly defined in the study.
10	McMahon SA et al.	Mongoro Region, Tanzania. The concept of “safe motherhood” was expanded to include not only mortality and morbidity but also human rights.	112 Individual interviews of mothers, male partners within 14 months of birth, public opinion leaders and community health workers.	Proving questions revealed significant reports of disrespect and abuse for which acquiescence and passivity were adopted as coping strategies. Males paid bribes, made formal complaints or resorted to aggression in response to the disrespect, abuse and neglect.
11	Miller S et al.	Domincan Republic, 14 facilities,	Facility assessment (chart review, interviews, observations compared to international RMC and clinical norms, facility statistics reviewed and compared with national statistics).	Maternal mortality was contributed to by provider attitudes, poor clinical skills, neglect of patients with the two tiered fee structure in the healthcare system (private and public), poor staffing, lack of emergency skills training.
12	Mselle LT et al.	Sample from Comprehensive Community Based Rehabilitation in Temeke district hospital in Dar es Salaam, and Mpwapwa district in Dodoma region of Tanzania. This hospital manages 26, 568 births annually and one of two specialty hospitals for fistula surgery. Patients come from both Mpawapwa district and Temeke district.	Semi-structured interviews with 16 mothers diagnosed with obstetric fistula, 5 nurse-midwives and focus group discussions with husbands and community members	Women described mothers labouring and birthing without attendance, lack of support, lack of equipment and service, physical and verbal abuse. Providers reported lack of supportive supervision, staffing and supplies. Nurse-midwives experienced lack of motivation, disempowerment and moral distress. This is one of the few studies to describe the self-reports and work-life experiences of Tanzanian midwives.
13	Penfold S et al.	The study was part of INSIST in six districts of Lindi and Mtwara regions, Tanzania, (population 1,000,000 in 2007)	Mixed method study (cross-sectional survey of all health facilities in the study districts, and qualitative focus group discussions and interviews with health managers)	200 facilities were sampled. Staff reported lack of essential medications, equipment shortages, and glove shortages. They coped by adapting or improvising clinical care. Logistics problems were not explored to determine the causes of the poor drug and equipment supplies.
14	Ratcliffe HL et al.	Large referral hospital in Dar es Salaam, Tanzania with a catchment of 1.4 million people.	Exploratory study. 2000 women were interviewed immediately after birth, 77 of which were also interviewed 4–6 week postpartum. Staff was also monitored following RMC interventions in the facility. Patients evaluated and community interviews conducted after participating in RMC initiatives such as Open Birth Days (similar to an open house)	Average age oft the women was 29.7 yr. 10% were HIV positive, 17.5% nulliparous. 82.6% married with primary education or greater. 15% reported experiencing any category of disrespect and abuse. The staff were given RMC training which providers. The facility held Open Birth Days, which patients found helpful in improving their knowledge and expectations for birth.
15	Rosen HE et al.	Tanzania was one of 5 countries studied (others included Kenya, Madagascar, Rwanda). In Tanzania, 52 facilities, 12 hospital, and 40 centers were sampled.	Structured, standardized clinical observations, cross sectional surveys from 2009 to 2012 as part of the Maternal and Child Health Integrated Program to assess quality of care using a checklist.	2164 labour and birth observations occurred in 1458 patients at hospitals, dispensaries and health centres. 320 of the clients were from Tanzania. Providers (doctors, nurse-midwives, students) obtained informed consent and refusal 62% of the time. Dignity was demonstrated with a friendly greeting 94.6% of the time. Encouragement to have a labour support person only occurred 39.5% of the time. Explanation of procedures occurred 72.1% of the time. Asking if the client had questions only 26.8% of the time. Respect for the woman’s choices was often not observed. Permitted to ambulate only 54.8% of the time. Draping before delivery occurred only 46.1% and visual privacy occurred only 35% of the time in a shared room. However, 93.2% of the time the provider provided friendly support during labour. Although small in sample size, the findings support other larger studies.
16	Sando D et al.	Large urban regional referral hospital in Dar es Salaam, Tanzania. The Labour unit is often crowded with patients and understaffed. There is significant stigma in Tanzania attached to HIV positive status.	Mixed methods (interviews of 2000 postpartum women, direct observation 208 births, 50 structured questionnaires and 18 in-depth interviews	12.2% of the HIV positive participants and 15% of the HIV negative participants reported disrespect and abuse during childbirth (*p* = 0.37). These came in the form of abusive statements and non-consented care. None of the participants reported violations of confidentiality in terms of the HIV status. Staff reported shortages of gloves, cotton wool and overcrowding to be constant challenges but appeared to be engaging in the norms and standards to prevent mother to child transmission of HIV. Despite the size of the hospital and the staff shortages, HIV positive mothers were receiving adequate health education.

### Conceptualization of respectful care

Respectful care is emerging as a phenomenon of study since the World Health Organization (WHO) conceptualization was published in 2015 [4]. The White Ribbon Alliance operationalized the conclusions of the WHO into seven domains or standards of disrespectful care in the Universal Rights of Childbearing Women. Across the RMC literature reviewed, the authors found wide acceptance of these categories.

While there is some early foundational work on RMC dating back to the early 2000s [5], a majority of the literature reviewed was published after 2010 (See Table 2). We noted a rising spike in publications on this topic, including the WHO conceptualization in 2015 and a Lancet special issue in 2016 [4, 6]. As might be expected for a new field of research, much of the literature reflects the preliminary nature of our understanding of RMC. Investigators for case and field studies such as Rosen et al. [7] noted that study designs were pilots and much of the work sought to solidify and validate the domains of RMC. A more recent systematic review by Shakibazadeh et al. confirms previous reviews that capture the global nature of disrespect and abuse; but they go further to identify a total of 12 relational and infrastructural domains, thereby making the facility managers just as culpable in disrespect and abuse. [8] Of note, there are a growing number of observational, descriptive and mixed methods RMC studies specific to Tanzania. [6, 9–15].

We note that although disrespectful care is observed worldwide, in both the Global North as well as the Global South, disrespectful care by maternal care providers has been studied primarily in low-resource countries such as the Dominican Republic [16], India [5, 17], Kenya [18, 19], Peru, Burundi [5], Nigeria, and Tanzania [5, 7, 19, 20].

Work documenting disrespectful care contributes to an overall understanding of the subject and suggests methods for quantifying and comprehending the scale of disrespectful care, a first step to combating it.

There is also a growing body of work suggesting and evaluating interventions to improve the quality of care and identifying the conditions that promote respectful care but little in the way of evidence-based clinical guidelines [6]. In contrast, the systematic review by Prost et al. [21] and the study by Bhutta et al. [22] demonstrate that interventions such as deploying community workers who are skilled in cultural sensitivity and respectful communication strategies might improve maternal satisfaction, service utilization and perinatal outcomes in Pakistan. In a mixed method study that included 52 Tanzanian facilities, Rosen et al. [7] observed that insufficient communication and information sharing by providers, delays in care, abandonment of laboring women, lack of a patient-centered approach by hospital administration and poor infrastructure contributed to disrespectful care (See Table 3).

**Table 3:** Summary of RMC methodologies and frameworks

Type of RMC Research Question	Quantitative	Theoretical Framework
	Methodology	
Do health education interventions improve facility utilization?	Quasi experimental before-and-after	Community-based participatory framework
Does disrespect and abuse correlate with facility utilization?	Correlational	Not usually stated
What is the incidence and prevalence of disrespectful care and abuse? What types of abuse occur? What are the perinatal outcomes/indicators in the facilities where disrespect and abuse occur?	National health surveys Institutional surveys National household surveys Demographic health surveys Facility statistics Population surveys/epidemiological surveys	RMC Medical models Public health models
Describe the elements of disrespect and abuse	Case control studies Prospective closed cohort	Critical human rights, reproductive rights
Do RMC-related community interventions improve perinatal outcomes? (deploying Lady Health or Community Health Workers)	Clustered RCT	Medical model WHO and MDG focus
Does strengthening one or more domains of RMC affect perinatal outcomes?	Correlational Retrospective descriptive Self-administered surveys	Medical model Public health models
	Qualitative	
What do providers and families identify as important to quality, satisfying maternity care and desirable healthcare work environment?	Exploratory	Critical Human Rights, reproductive rights
Compare client’s lived experience of respectful versus disrespectful care	Phenomenological hermeneutics with semi-structured interviews	Human rights Childbirth Rights Critical Social Theory
Describe the work-life experience of the healthcare workers when disrespect and abuse are occurring. Describe the lived experience of vulnerable groups when disrespect, abuse or RMC occurs.	Focus groups	Resilience theory
What are the barriers to provision of RMC and what are the recommendations of midwives for improving care quality?	Individual and focus group interviews	RMC Feminist version of post-structural interactionism
	Mixed Methods	
What types of abuse occur? Which providers perpetrate abuse? (MD, RN, RM, resident, staff MD, student midwife) What are their number of years of professional training, years of practice, amount of RMC training?	Institutional/rapid Assessment (including 2-person expert observations, surveys, focus groups, semi-structured individual interviews of staff and patients, facility check-lists based upon national professional standards and WHO standards, facility statistics MMR, IMR)	RMC Human rights Childbirth rights

*Abbreviations*: *MMR* Maternal mortality ratio, *IMR* Infant mortality rate, *RMC* Respectful maternity care, *MDG* Millenium Development Goals, *RCT* Randomized controlled trial, *WHO* World Health Organization

Many authors note that disrespectful care is not well-studied, nor is it well-recorded, and there are methodological challenges to conducting this research [23]. If each of the RMC domains is treated as categorical or dichotomous variable to describe its effect either on facility utilization or on perinatal outcomes, separating the effects of changes to each variable has yet to be designed into logistic regression models. Yet, research utilizing trained community health workers or respectful centering pregnancy models do not control for facility or infrastructure factors. Notwithstanding, there may be ethical dilemmas in purposely eliminating one of the seven factors in favour of examining another. Consequently, though constituting a higher order of evidence [24, 25], randomized control trials (RCTs) are problematic.

This leaves us with observational, retrospective, case studies, and various qualitative studies to understand why midwives or mothers define some care as dignified or respectful while finding other types of care disrespectful. Women may be reluctant to share experiences of disrespectful care. One author notes that women may not divulge disrespectful care and that the language with which women describe disrespectful care may go unrecognized [26]. For example, in a qualitative study the women often responded positively when first asked about their birth experience generally. However, more careful probing would often reveal disrespectful care (such as “screaming” or “speaking roughly”). This underscores the necessity for a tangible definition of respectful care, such is provided in the seven domains, to allow for uniform documentation [13].

Table 2 summarizes methodologies used to examine all or some of the domains of respectful maternity care in the research reviewed between the years of 2000 and 2016. This foundational research has provided an excellent springboard for action research and quality improvement evaluation following RMC interventions. In the years to come, healthcare providers, policy makers and educators should anticipate curriculum development and post-service training to be informed by the emerging quality improvement research linked to RMC. Within these studies, vulnerable populations have been sampled, such as adolescent mothers [19, 27], mothers who are HIV positive [19, 20], mothers of lower socioeconomic status, discriminated ethnic groups or castes [17, 26], and new immigrant mothers [28], with particular emphasis on their experience of discriminatory beliefs, attitudes and practices [20].

Ratcliff et al. [6] describes the use of an educational program and workshop that involved strengthening many skills in two Tanzanian facilities. The aspects addressed included birth preparedness skills, patient-provider communication and provider-administrator communication skills. They found that patients reported increased feelings of empowerment and confidence during delivery. Providers reported increased job satisfaction and improved quality of care was recorded by external observers. Many researchers emphasize the need for RMC provider training which includes strategies for communication with the hospital administration regarding infrastructure and staffing needs as key elements [29].

A critical analysis of the literature reveals that increased access to high quality care will not necessarily improve outcomes without community engagement. Previously, global development was focused on increasing the numbers of facilities, the equipment, the numbers of providers, and modes of transportation, presumably to improve access to care [13, 20, 30–32]. However, mothers continued to avoid care due to disrespectful behaviors of the caregivers [18]. Mothers desired and/or were denied adequate informed consent [18, 19, 28, 33, 34]. They report that the provider failed to include them in decision-making process surrounding admission and plan of care. They report that they had little understanding of rationale for interventions [19, 34, 35]. It would be unfair to simply draw conclusions for a causative relationship between disrespectful care and lack of skill amongst providers. However, by hearing the lived work experiences of midwives in the Global South, valuable data has begun to emerge that indicate disrespectful care is a multifactorial phenomenon. Consequently, education of midwives solely, without changing the conditions of midwifery work might prove to be ineffective.

### The work-life of the midwife

Researchers are attempting to document the conditions from which disrespectful care emerges. Midwives in the Global South described challenging shortages of equipment and staff [19, 36]. They also expressed job dissatisfaction, low morale or motivation, significant desire to quit and inadequate training [34, 36–38]. The researchers cited these issues as barriers to caring for women adequately and deterrents to accessing care. Researchers explain that this weak infrastructure discourages respectful care [19, 36, 38]. (These studies also describe higher incidences of adverse outcomes such as maternal and newborn deaths).

Possibly, midwives who describe a sense of oppression or constraint due to the public and facility policies within which they work, may also be less likely to work efficiently or respectfully [39]. Therefore, rather than a punitive, oppressive approach, educators, researchers and policy-makers have addressed disrespectful care by building human resource capacity, by strengthening professional organizations and by educating midwives in low-resource countries. Furthermore, researchers such as Ratcliff et al. [6] encourage midwives not only to acquire attitudinal change and to adopt respectful maternity care skills, but also to emerge as leaders who challenge policy-makers, institutional administrators and politicians to strengthen the healthcare system and infrastructure that effects respectful maternity care so significantly. Notwithstanding, the process of becoming a change agent is not easy in the Tanzanian context due to the organizational system and culture, where midwives are so immersed in the work of midwifery that they describe being less informed and unable to advocate for themselves regarding their work-life. The next steps in research will require evaluation of the RMC strategies and interventions that have been employed since the 2015 challenges posed by the WHO, preferably including the perspectives of midwives [4].

### Leadership and change

It is clear from a few Tanzanian studies that the midwives were attempting to address severe and urgent crises. In fact, Penfold and colleagues [29] noted that distressed staff in Tanzanian facilities coped with the unsatisfactory working conditions by dangerous risk-taking behaviours; including improvisation in the absence of functioning equipment or sufficient supplies, alternative forms of sterilization that are not evidence-based and shorten the life of the equipment, risking their own health and safety by avoiding infection control standards to perform life-saving procedures for patients (e.g., mouth-to-mouth resuscitation for newborns). It is unknown whether their patients or families received these efforts positively, and whether these efforts mitigated some of the perceptions of disrespect that reportedly deter families from using healthcare facilities [14]. Consequently, safe methods of addressing maternal and newborn mortality and morbidity need to be developed. Emerging evidence points to interventions such as education in crisis management, leadership and communication as integral to RMC training.

Clearly action research needs to measure the outcomes of clinical and social innovations employed in the low-middle-income countries (See Table 4). These innovations were in both Tanzania and South Sudan by some of the authors. Qualitative, substantive changes such as creating privacy drapes, using temporary privacy walls with drapes on intravenous poles, obtaining informed consent and refusal. The younger participants with 3-year diplomas and 4-year degrees tended to generate more innovative digital solutions such as an electronic form of the WHO mandated Partograph [40], however these creative ideas require start-up grants and major global health funding to implement.

**Table 4:** Social and clinical innovations

Social or Clinical Innovations Recommended	Organization who has Recommended These Interventions
Open maternity days or open houses	Population Council
Provider debriefing and psychosocial support	Population Council
Redevelop partograph	WRA adaptation as an RMC eval tool, UNFPA recommended modifications, Jhpiego e-partograph (https://bmcpregnancychildbirth.biomedcentral.com/articles/10.1186/s12884-018-1760-y)
Mediation program	Population Council
Respectful Maternity Care training workshop	WRA Population Council TAMA/CAM/Jhpiego partnership
Health Facility Management Board or Multidisciplinary Stakeholders group (politicians, business, legal council, writers, journalists)	Population Council Midwives in TAMA RMC Workshops
Elders meetings and community engagement strategies (teas)	Midwives in TAMA RMC Workshops
Emergency Skills Workshops infused with RMC PBL	Midwives in TAMA RMC Workshops
Mediators appointed from laws school students, retired lawyers, social workers	Midwives in TAMA RMC Workshops
Anteroom outside of delivery room where families may verbally provide ongoing support to birthing/laboring mother	CEPBU Community Health Centres, Burundi http://fr.allafrica.com/stories/200703020713.html
Educate more mother/family friendly allies and champion in the professional community	NGO “Save the Mothers” founded an interdisciplinary Master of Public Health Leadership in Uganda. https://www.savethemothers.org/what-we-do/degree-program/

*Abbreviations*: *CAM* Canadian Association of Midwives, *CEPBU* La Communauté des Eglises de Pentecôte du Burundi, *NGO* Non-governmental organization, *PBL* Problem-based learning, *TAMA* Tanzanian Midwives Association, *UNFPA* United National Population Fund, *WRA* White Ribbon Alliance

The participants of the RMC workshops requested ongoing continuing education in RMC. They found the workshops cathartic for those who suffered post-traumatic grief after having engaged in or witnessed disrespect and abuse. They also found the workshops to be synergistic and empowering in terms of findings practical, low-cost, effective solutions to complex social situations, complicated by low resources and high risk clinical decision-making. Recommendations from the participants and consultants were to meet with ministry of health professionals, local midwifery educators and midwife preceptors in social innovation rounds to discuss common goals and troubleshoot for solutions over an interprofessional, informal gathering. In countries rife with high context cultural norms where meetings must be officiated by respected high ranking medical officers or government representatives, these meetings are essential if RMC initiatives are to be endorsed and ratified. Similarly, student midwives learning to apply evidence-informed care need the support of effective midwife allies who will model leadership and courage as they advocate for respectful care of vulnerable clients and culturally safe engagement with the community.

## Conclusion

Defining disrespectful care in a tangible way with concrete examples will aid in research and intervention design, as well as the sharing of best practices and interventions. A general terminology and taxonomy of RMC has clearly emerged over the past two decades. This is facilitating knowledge exchanges, and it is also helping to aid researchers and practitioners to gain resources which promote RMC.

However, within this broader understanding of how RMC impacts birth outcomes and maternal mortality, we note the importance of centering the birthing person and their inherent *dignity in its own right*, regardless of the outcomes, lest we exploit respectful care as a fleeting ploy to pacify hospital administrators. We noted that most studies linked disrespectful care to low uptake of skilled birth attendance and negative health outcomes. It is therefore essential to speak of both the impact on birth outcomes (including mortality), as well as the important personal and individual lived experience of the birthing mother. Importantly, it is the experience that the birthing mother brings (biological, ancestral, and lived) that are undermined when personal choice is not respected in the birthing environment (See Table 1, Domain 4). We believe that most authors recognize this implicitly, however, it is worth articulating and repeating.

Table 2 describes the various types of research questions that define the RMC problems faced by healthcare providers in low-resource countries. Future research questions need to measure the effectiveness of interventions directed at all seven domains of RMC. Researchers posit that ongoing structural, attitudinal and healthcare system changes will significantly affect facility utilization, perinatal outcomes, healthcare provider retention and the overall quality of maternity care in the Global South [5, 18, 28]. Tanzania, the recipient of many global development grants, is poised to pilot many of the recommendations emerging from the research (See Table 2).

## Abbreviations

CEPBU: La Communauté des Eglises de Pentecôte du Burundi
IMR: Infant mortality rate
MDG: Millenium Development Goals
MMR: Maternal mortality ratio
PBL: Problem-based learning
RCT: Randomized controlled trial
RMC: Respectful maternity care
TAMA: Tanzania Midwives Association
WHO: World Health Organization
